# YAP Inhibition by Verteporfin Causes Downregulation of Desmosomal Genes and Proteins Leading to the Disintegration of Intercellular Junctions

**DOI:** 10.3390/life12060792

**Published:** 2022-05-26

**Authors:** Yunying Huang, Usama Sharif Ahmad, Ambreen Rehman, Jutamas Uttagomol, Hong Wan

**Affiliations:** Centre for Oral Immunobiology and Regenerative Medicine, Institute of Dentistry, Barts and The London, School of Medicine and Dentistry, Queen Mary University of London, London E1 2AT, UK; yunying.huang@qmul.ac.uk (Y.H.); u.s.ahmad@qmul.ac.uk (U.S.A.); ambreen.rehman@duhs.edu.pk (A.R.); jutamasu@nu.ac.th (J.U.)

**Keywords:** verteporfin, YAP, desmoglein 3, desmosomes, junction formation, keratinocyte

## Abstract

The Hippo-YAP pathway serves as a central signalling hub in epithelial tissue generation and homeostasis. Yes-associated protein (YAP) is an essential downstream transcription cofactor of this pathway, with its activity being negatively regulated by Hippo kinase-mediated phosphorylation, leading to its cytoplasmic translocation or degradation. Our recent study showed phospho-YAP complexes with Desmoglein-3 (Dsg3), the desmosomal cadherin known to be required for junction assembly and cell–cell adhesion. In this study, we show that YAP inhibition by Verteporfin (VP) caused a significant downregulation of desmosomal genes and a remarkable reduction in desmosomal proteins, including the Dsg3/phospho-YAP complex, resulting in attenuation of cell cohesion. We also found the desmosomal genes, along with E-cadherin, were the YAP-TEAD transcriptional targets and Dsg3 regulated key Hippo components, including *WWTR1*/TAZ, *LATS2* and the key desmosomal molecules. Furthermore, Dsg3 and phospho-YAP exhibited coordinated regulation in response to varied cell densities and culture durations. Overexpression of Dsg3 could compensate for VP mediated loss of adhesion components and proper architecture of cell junctions. Thus, our findings suggest that Dsg3 plays a crucial role in the Hippo network and regulates junction configuration via complexing with phospho-YAP.

## 1. Introduction

The Yes-associated protein (YAP) is a transcription cofactor and acts as a key downstream effector of the Hippo signalling pathway, an evolutionarily conserved network that plays a central role in the maintenance of tissue integrity and homeostasis [1,2,3]. The Hippo-YAP pathway regulates a diversity of cellular processes, such as cell proliferation, differentiation, migration, survival and anti-apoptosis [2,4]. This pathway also functions as a sensor and mediator of many extracellular and intracellular signals, including that mediated by reactive oxygen species (ROS) [5,6]. The activity of YAP is negatively regulated by phosphorylation via upstream Hippo kinases leading to its nuclear exclusion and cytoplasmic accumulation or degradation [2,7]. Overexpression of YAP stimulates cell proliferation, whereas depletion of YAP or treating cells with its inhibitor Verteporfin results in an inverse effect, even in the presence of growth factors [8,9]. YAP is also regulated by Hippo-independent mechanisms [3]. Since YAP lacks DNA-binding activity its transcriptional function is via interacting with DNA-binding transcription factors such as TEAD that together form the YAP-TEAD complex regulating the target gene’s expression [10]. Therefore, YAP may act either as an oncogene or tumour suppressor depending on its binding partners. Dysregulation of the Hippo-YAP pathway can lead to an array of diseases, including cancer and skin blistering conditions such as pemphigus [3,5,11,12].

Our recent studies have shown that the desmosomal cadherin Desmoglein-3 (Dsg3) is capable of regulating YAP expression and sequesters preferentially phospho-YAP S127 (p-YAP) to the cell surface, presumably to facilitate epithelial cell junction formation [13]. In line with these findings, overexpression of Dsg3-induced p-YAP expression and increased YAP luciferase activity as a consequence of repressing collective migration in oral keratinocytes [14]. On the other hand, YAP depletion evokes upregulation of adhesion molecules, such as Dsg3, α-Catenin, and plakophilins 1/3 and promotes junction formation as well as its structural integrity [5,14]. Hence, it was plausible that a similar effect would be observed by YAP inhibition that could lead to augmented expression of junction assembly proteins and enhanced intercellular adhesion. However, there remains little evidence in the literature about the biological consequence of YAP inhibition in cell junction adhesion and integrity, in particular in the context of desmosomes.

Verteporfin (VP) is a photosensitizer used in photodynamic therapy with limited toxicity [15]. Recently, VP has been identified as an inhibitor of YAP, without photoactivation, via blocking the interaction of YAP with TEAD, which, in turn, blocks transcriptional activation of YAP downstream targets [16,17]. Treating cells with VP also results in a decrease in both basal and EGF-induced YAP nuclear localisation and concurrently, induces YAP cytoplasmic translocation through increasing expression levels of 14-3-3σ or targeting YAP degradation in the proteasome [9]. Thus, increasing interest has been drawn in utilising VP as a promising anti-cancer chemotherapeutic and adjuvant drug [8,18,19,20,21,22]. Although VP is known to have an impact on cell survival and apoptosis, the effect of VP on cell junctions, such as desmosome stability, had not yet been explored. This study aimed to investigate the impact of VP-induced YAP inhibition on cell junction formation, structural integrity and cohesion. The results from this study shed light on the vital role of YAP in epithelial cell junction arrangement and indicated that VP mediated YAP inhibition can abolish cell junctions (that differs from what we initially anticipated), caused by pronounced alterations in the stability of adhesion proteins and cell junction attenuation. Importantly, the study uncovered that the desmosomal genes, as well as E-cadherin, are the targets of YAP-TEAD nuclear transcriptional activity with Dsg3 playing a vital role in controlling the balance of the Hippo network.

## 2. Materials and Methods

### 2.1. Antibodies

The following mouse and rabbit monoclonal/polyclonal antibodies (Abs) were used: D8H1X, rabbit Ab to YAP (Cell Signaling Technology, Danvers, MA, USA); Anti-YAP1 (phospho S127) antibody (Abcam, Cambridge, MA, USA); YAP/TAZ (D24E4) Rabbit mAb (Cell Signaling Technology, Danvers, MA, USA); 5H10, mouse Ab against the N-terminus of Dsg3 (Santa Cruz, Santa Cruz, CA, USA); 33–3D, mouse IgM against Dsg2 (gift from Professor Garrod); PG 5.1, mouse Ab to Plakoglobin (Progen, Heidelberg, Germany); 115F, mouse Ab to Desmoplakin (gift from Professor Garrod); H-300, rabbit Ab to Desmoplakin (Santa Cruz, Santa Cruz, CA, USA); 5C2, mouse Ab to plakophilin1 (Progen, Heidelberg, Germany); PKP3, mouse Ab (Abcam, Cambridge, MA, USA); HECD-1, mouse anti-N-terminus of E-cadherin (Abcam, Cambridge, MA, USA); rabbit Ab to α-catenin (Abcam, Cambridge, MA, USA); 6F9, mouse anti-β-catenin ascites fluid (Sigma, St. Louis, MO, USA); mouse Ab to K14 (gift from Professor Leigh); 14C10, rabbit Ab to Glyceraldehyde-3-phosphate dehydrogenase (GAPDH)-Loading control (Cell Signaling Technology, Danvers, MA, USA); Secondary Abs were Alexa Fluor 488 goat anti-mouse/rabbit IgG (Invitrogen, Waltham, MA, USA) and Alexa Fluor 568 goat anti-mouse/rabbit IgG (ThermoFisher Scientific, Waltham, MA, USA) or horse radish peroxidase goat anti-mouse/rabbit IgG (Sigma, St. Louis, MO, USA), respectively.

### 2.2. Keratinocyte Culture and Treatment

The immortalized normal skin-derived human keratinocyte line N/TERT was maintained in keratinocyte serum-free medium (KSFM) (ThermoFisher Scientific, Waltham, MA, USA) as described previously [5,23]. T8 cutaneous squamous cell carcinoma cell line (gift from Prof. Catherine Harwood) was cultured in a complete keratinocyte growth medium (KGM containing Dulbecco’s Modified Eagle Medium (DMEM) (Lonza, Basel, Switzerland): Ham’s F-12 (ThermoFisher Scientific, Waltham, MA, USA) in the ratio of 3:1 supplemented with 10% fetal calf serum (FCS) (Biosera, San Diego, CA, USA), epidermal growth factor (EGF) (Invitrogen, Waltham, MA, USA), insulin human solution (Sigma, St. Louis, MO, USA), cholera toxin (Sigma, St. Louis, MO, USA), and hydrocortisone (Sigma, St. Louis, MO, USA) [23]. Stable T8 cell lines with hDsg3.myc (D3) and the matched empty vector control line (Vect) were generated in this laboratory following the procedures described previously [24,25]. For the dose and time-course experiments with verteporfin (VP), cells were seeded at approximately 70~80% confluent densities and cultured in KGM [23] in the presence and absence of VP at various concentrations (with a range of dosages similar or less than others [8,9,26]) for different time frames before analyses by different techniques and assays, respectively.

### 2.3. MTT Assay

The 3-(4,5-dimethylthiazol-2-yl)-2,5-diphenyltetrazolium bromide (MTT) assay was used to assess the effect of VP treatment on the cell viability. Briefly, cells were seeded at approximately 70~80% confluence in triplicates in a 96-well plate overnight before being treated with VP at various concentrations, i.e., 1, 3 and 5 µg/mL for 2, 6 and 24 h, respectively. Then, 100 µL MTT was added to each well and incubated for 4 h at 37 °C. After removing the media, 150 µL dimethyl sulfoxide was added to dissolve the formazan crystal and optical density was measured using a plate reader at 570 nm.

### 2.4. Trypan Blue Assay

The trypan blue assay was performed by mixing 1 part of 0.4% trypan blue with 1 part cell suspension of VP treated cells and allowing the cell mixture to incubate for 3∼5 min at room temperature. Then, both the unstained (viable) and stained (nonviable) cells were separately counted with a haemocytometer. The total numbers of viable and nonviable cells per ml were calculated by multiplying the total number of cells by 2 (the dilution factor for trypan blue), respectively, and presented as a percentage of trypan blue negative cells in a graph.

### 2.5. Measurement of Reactive Oxygen Species 

The cellular reactive oxygen species (ROS) levels were determined by incubating cells in 96-well plates with CellROX Oxidative Stress Reagents (Molecular Probe by Life Technologies, Carlsbad, CA, USA) at a final concentration of 5 µM for 30 min at 37 °C [5] before washing in PBS briefly. Live-cell image acquisition was carried out with a 20× objective using the INCA 2200 imaging system (GE Healthcare, Chicago, IL, USA).

### 2.6. Dsg3 siRNA Transfection

Transient Dsg3 siRNA transfection was performed in N/TERT cells as described previously [13,23]. A custom designed siRNA sequence (referred to as RNAi) specific for human *DSG3* mRNA, corresponding to nucleotides 620 to 640 of the respective coding region (Accession: NM_001944.1) (AAATGCCACAGATGCAGATGA) was synthesized by Dharmacon Research (Lafayette, LA, USA) as described previously [13]. In addition, another siRNA sequence was purchased from Dharmacon (ON-TARGETplus J-011646-05 referred to as siRNA-2), plus a scrambled control siRNA of a randomised version of the RNAi sequence. In brief, the transfection of siRNA was performed by seeding 2 × 10^5^ cells in a 6-well plate overnight before siRNA transfection at a final concentration of 50 nM using DharmaFECT 1 (Dharmacon, Lafayette, LA, USA) following the manufacturer’s instructions. The next day, cells were harvested with 0.25% Trypsin/EDTA and re-plated for various assays or analyses by different techniques.

### 2.7. Luciferase Assay

YAP/TAZ luciferase assay was performed as described in our previous report [23], using the Bio-Glo™ Luciferase Assay System (Promega, Madison, WI, USA) according to the manufacturer’s instructions. Briefly, T8 cell lines were seeded at 1 × 10^5^ in a 24-well plate overnight before transfection with 0.25 μg YAP/TAZ luciferase reporter plasmid [27] (Plasmid #34615, Addgene, Watertown, MA, USA) per well using FuGENE^®^ HD Transfection Reagent (Promega, Madison, WI, USA). After 24 h, cells were washed twice with PBS before the luciferase assay.

### 2.8. Dispase Dissociation Assay

The cell–cell adhesion was analysed by the dispase dissociation assay [28]. Briefly, cells were seeded at confluent densities (2 × 10^6^ cells) in 6-well plates and grew for at least 2–3 days before being washed briefly with PBS followed by incubation in 2 mL HBSS (14025092, ThermoFisher Scientific, Waltham, MA, USA) containing dispase II (2.4 U/mL, ThermoFisher Scientific, Waltham, MA, USA]) for about 20 min at 37 °C in 5% CO_2_/ 95% air until the epithelial sheets released from the substrate. Then, the epithelial sheets were washed with PBS twice before being subjected to pipetting with a 1 mL pipette at equal times for all samples to break the epithelial sheets. Fragments in each well were imaged using the ChemiDoc MP Imaging System (Bio-Rad) with Image Lab Software (Version 5.0, Bio-Rad Laboratories, Hercules, CA, USA). Finally, the number of fragments for each sample was determined with ImageJ software.

### 2.9. Quantitative Reverse Transcription-Polymerase Chain Reaction (RT-qPCR)

This method was described elsewhere in a previous study [13,14]. Briefly, mRNA was extracted using the Dynabeads™ mRNA DIRECT™ Purification Kit (ThermoFisher Scientific, Waltham, MA, USA) and was converted to cDNA using the qPCRBIO cDNA Synthesis Kit (PCR Biosystems, London, UK). RT-qPCR was performed using the qPCRBIO SyGreen Blue Mix Lo-ROX (PCR Biosystems, London, UK) in the LightCycler^®^ 480 Multiwell Plate 384 (Roche Life Science, Basel, Switzerland). The thermocycling begins with 95 °C for 30 s prior to 45 cycles of amplification at 95 °C for 1 s, 60 °C for 1 s, 72 °C for 6 s, 76 °C for 1 s (data acquisition). A “touch-down” annealing temperature intervention (66 °C starting temperature with a stepwise reduction of 0.6 °C/ cycle; 8 cycles) was introduced prior to the amplification step to maximise primer specificity. Melting analysis (95 °C for 30 s, 65 °C for 30 s, 65–99 °C at a ramp rate of 0.11 °C/s) was performed at the end of qPCR amplification to validate single product amplification in each well. The advanced relative quantification of mRNA expression was calculated based on an objective method using the second derivative maximum algorithm using the LightCycler^®^ 480 Software (Roche Life Science, Basel, Switzerland). *B2M* and *POLR2A* were used as stable reference genes to normalise all target genes. Each sample was run in quadruplicates as a routine. The primer sequences for all genes analysed in this study were published in our previous studies [13,14].

### 2.10. Immunofluorescence (IMF) and Image Analysis

IMF was performed in cells seeded either on coverslips or 96-well plates. Cells were fixed with 3.6% formaldehyde for 10 min, followed by cell membrane permeabilization with 0.1% Triton X-100 in PBS for 5 min [13,14], as a routine unless otherwise stated. The nonspecific binding sites were blocked for 15–30 min with 10% goat serum (Sigma, St. Louis, MO, USA) before the primary and then the secondary antibody incubations, each lasting for 1 h at room temperature (RT). Cells were washed 3 times with PBS containing 0.2% Tween 20 after each antibody incubation. Finally, coverslips were counterstained with DAPI for 8–10 min before a final wash and mounted on slides. For 96-well plates, cells were subjected to counterstain with DAPI and HCS Cellmask Deep Red stain (Invitrogen, Waltham, MA, USA) for 120 min before 3X PBS washing and image acquisition with a 20× objective using the INCA 2200 imaging system (GE Healthcare, Chicago, IL, USA) and 16 images were acquired per well as a routine. The fluorescence intensities were determined using the Developer Toolbox software (GE Healthcare, Chicago, IL, USA). For each antibody staining on coverslips, 5~6 images were acquired as a routine with a 40× objective using a Leica DM4000 Epi-Fluorescence microscope or a 63× oil objective using a Zeiss 710 Laser Scanning Confocal Microscope. Image quantification was determined with ImageJ (NIH Image, Bethesda, MD, USA). Finally, the mean IMF intensity per cell for each antibody staining was calculated by dividing the total IMF signals by the cell number per image before statistical analysis. Data were presented as the mean IMF intensities per cell for each condition in the graphs.

The co-immunoprecipitation and Western blotting analyses were performed as our routine as previously described [5,13,14,23].

### 2.11. Statistical Analysis

The results are presented as Mean ± SEM from repeated experiments unless otherwise stated. To quantify the IMF intensity of microscopic images, more than 300 cells in each sample were analysed as a routine. Normality of data distribution was checked with GraphPad software before one-way analysis of variance (ANOVA) analysis in multiple group comparisons or two-tailed Student’s *t*-test between two-group comparisons. *p* values of less than 0.05 was considered statistically significant, i.e., * *p* < 0.05, ** *p* < 0.01, *** *p* < 0.001, and **** *p* < 0.0001. Wherever possible, the comparison between control and test groups was normalized against the control (set as 1) and expressed as a fold change in the final plots.

## 3. Results

### 3.1. Dsg3 Forms a Complex with YAP and Phospho-YAP and Regulates Their Expression and Distribution in Normal Keratinocytes

**Figure 1:**
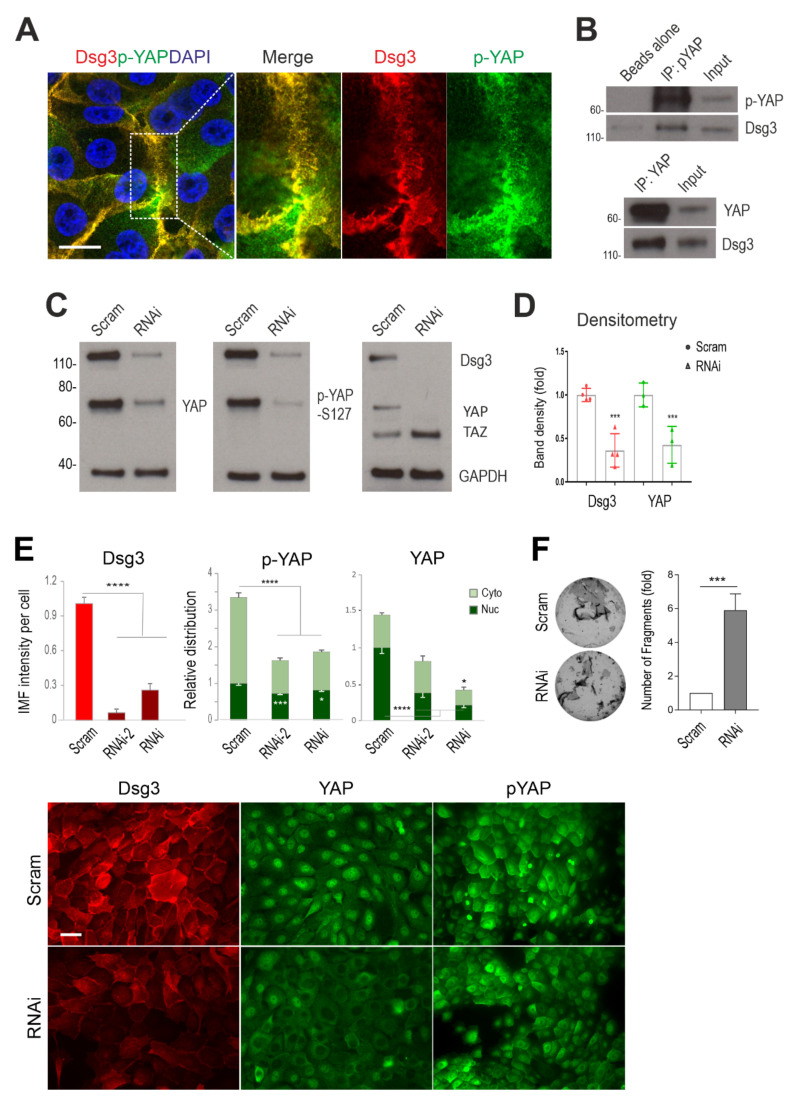
Dsg3 complexes with YAP/p-YAP, with its knockdown resulting in their significant reduction and disruption of intercellular adhesion in N/TERT cells. (**A**) Confocal super-resolution microscopy of N/TERT cells double-stained for Dsg3 and p-YAP showed their colocalisation, especially at cell borders. The enlarged dotted box for each channel is displayed on the right, respectively. (**B**) Co-immunoprecipitation (co-IP) in freshly confluent cell lysates with antibodies for p-YAP (IP: pYAP) or YAP (IP: YAP) that demonstrated Dsg3 physically interacted with the protein complexes purified with anti-p-YAP or YAP. The control lane was Beads alone, and input was lysates before IP (7~8%) (n = 2 independent experiments performed). (**C**) Western blotting for the indicated proteins in N/TERT cells pre-treated with Dsg3 specific or scrambled control siRNA for 2 days (n = 3 independent experiments). GAPDH was used as a loading control. (**D**) Densitometry for the indicated protein blots. (**E**) Image quantitation for the indicated proteins and their subcellular distribution in cells transfected with two hits of Dsg3 siRNA. A significant reduction in YAP/p-YAP was detected in cells with Dsg3 knockdown. The representative images for the indicated proteins in control and Dsg3 knockdown cells are displayed below the bar charts (n = 5 images/sample, representative of three independent experiments, Mean ± SD). (**F**) Dispase cell dissociation assay in N/TERTs with Dsg3 knockdown or control cells treated with scrambled siRNA. The siRNA pre-treated cells were pooled at confluent densities one day after siRNA transfection and were allowed to grow for 2 days before dispase treatment at 2.4 unit/mL until the epithelial cell sheets detached from the substrate, followed by mechanical stress to induce fragmentation. Images are displayed on the left and the quantitation of fragments is shown on the right (n = 4, Mean ± SD). (Student’s *t*-test for two-group comparison or one-way ANOVA for three group comparison, * *p* < 0.05, *** *p* < 0.001 and **** *p* < 0.0001). Scale bar in A, 10 µm and E, 20 µm.

Our previous study showed that Dsg3 colocalised and formed a complex with p-YAP in HaCaT keratinocytes [13]. To confirm this finding exists in normal keratinocytes, we performed immunofluorescence and co-immunoprecipitation assays in normal skin-derived keratinocyte line, N/TERT cells. Cells were seeded on coverslips and fixed before being double-stained for Dsg3 and p-YAP. Consistent with our earlier report [13], super-resolution confocal microscopy revealed a high degree of the colocalisation of these two proteins, with representation by the yellow/orange pixels, especially at the cell borders (Figure 1A). Immunostaining for YAP showed predominant localisation in the perinuclear region with some membrane distribution still being detectable (data not shown). In line with this result, co-immunoprecipitation in freshly confluent N/TERT cell lysates with the anti-p-YAP antibody detected Dsg3 in the immune-precipitate, confirming their complex formation in N/TERTs (Figure 1B). Additionally, Dsg3 also was detectable in the immune-precipitate purified with the anti-YAP antibody. These results thus indicated that Dsg3 forms a protein complex with both total and the phosphorylated form (S127) of YAP in normal keratinocytes.

To verify the role of Dsg3 in regulating YAP, a Dsg3 knockdown experiment was performed with a specific siRNA, alongside scrambled control siRNA, in N/TERTs. One day after siRNA transfection, cells were replated at approximately 70% confluence in a 6-well plate and the lysates were extracted the next day for Western blotting analysis. The result showed a significant reduction in YAP (>2-fold) and p-YAP, in Dsg3 knockdown cells compared to control samples (Figure 1C,D). A similar result with YAP reduction was also obtained with the antibody to both YAP/TAZ that indicated compensation by TAZ (a paralog of YAP) in cells with a reduction in YAP caused by Dsg3 depletion. This result suggests that Dsg3 silencing caused decreased protein expression or stability of YAP/p-YAP. Next, immunostaining for Dsg3, YAP/p-YAP was conducted in cells transfected with scrambled or specific Dsg3 siRNA, including two hits to determine protein distribution. Image quantitation revealed a consistent finding of YAP/p-YAP reduction following Dsg3 depletion in both the nucleus and cytoplasm as opposed to controls (Figure 1E). Furthermore, the impaired cell–cell adhesion was proved by the functional dispase assay that demonstrated compromised cell cohesion with augmented fragmentation of epithelial sheets in Dsg3 depleted cells compared to controls (Figure 1F). Together, these data reinforce our previous findings and suggest that Dsg3 regulates the expression and stabilization of YAP/p-YAP and sequesters p-YAP to the cell surface to facilitate epithelial junction formation and/or cell–cell adhesion.

**Figure 2:**
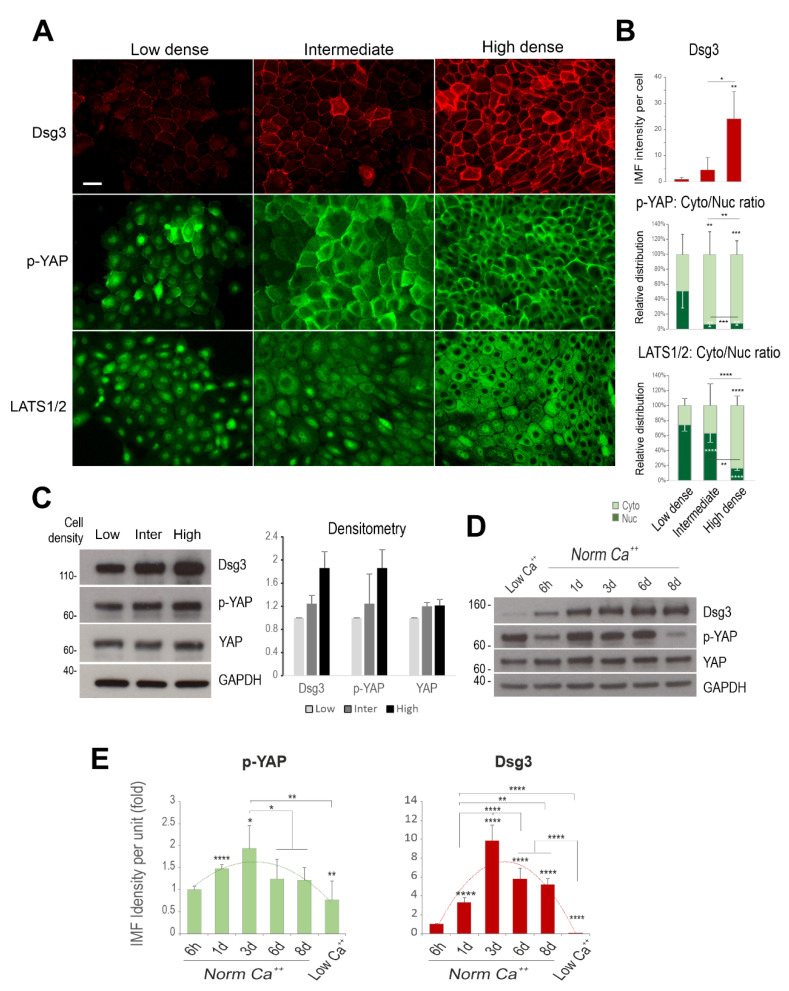
Coordinated regulation of Dsg3 and Hippo-YAP components in a cell density and a time-dependent manner. (**A**) Fluorescent images of N/TERT cells seeded at low, intermediate and high cell densities for 1 day before immunostaining for Dsg3 and p-YAP as well as Hippo kinase LATS1/2 showed a density-dependent increase in Dsg3 coupled with p-YAP/LATS1/2 nuclear exclusion, especially at the high cell density. (**B**) Quantification for the images shown in A. (**C**,**D**) Western blotting analysis of lysates extracted from cells grown at three different densities or in a time-course study that detected a cell density and time-dependent augmentation in both Dsg3 and p-YAP expression, with YAP levels appearing relatively stable, with the densitometry shown in C. For the time-course experiment, N/TERT cells were seeded in KSFM at low calcium (0.09 mM) before being replaced with KGM containing normal calcium concentration (1.8 mM). Cells were grown for various time frames before extraction or immunostaining for p-YAP and Dsg3 with the image quantitation shown in (**E**) and the representative images in Figure S1. A bell-shaped expression profile was detected for both p-YAP and Dsg3 (n = 5 images/coverslips, at least three experiments were performed, Mean ± SEM, *p* values were determined by one-way ANOVA, * *p* < 0.05, ** *p* < 0.01, *** *p* < 0.001 and **** *p* < 0.0001). Nuc: nucleus; Cyto: cytoplasm; Ca^++^: calcium ion. Scale bar, 20 µm.

**Figure 3:**
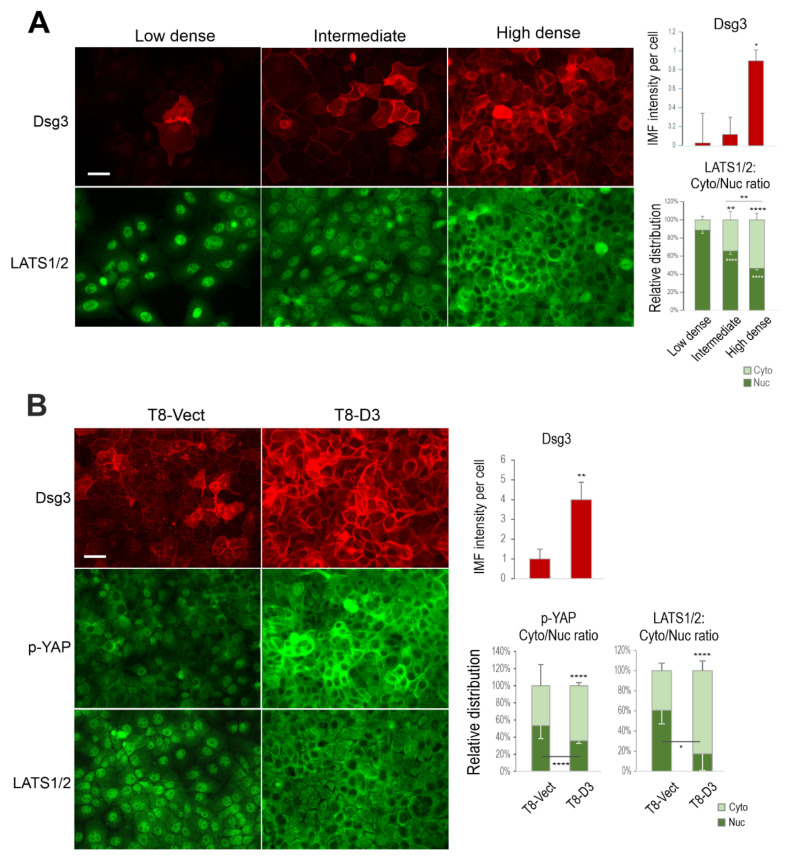
Coordinated regulation of Dsg3 and Hippo-YAP components in response to growing cell densities in T8 keratinocytes. (**A**) Fluorescent images of cutaneous carcinoma T8 (parental) cells seeded at low, intermediate and high cell densities for 1 day before immunostaining for Dsg3 and Hippo kinase LATS1/2 showed a density-dependent increase in Dsg3 coupled with LATS1/2 nuclear exclusion, especially at the high cell density. Image quantification for Dsg3 expression and LATS1/2 subcellular distribution was shown on the right. (**B**) Immunofluorescent staining for Dsg3, p-YAP and LATS1/2 in T8 stable lines with transduction of hDsg3.myc (D3) and the matched empty vector control line (Vect Ct) showed that the elevated Dsg3 expression was correlated with cytoplasmic translocation of p-YAP and LATS1/2, respectively. Image quantification for Dsg3 expression and p-YAP/LATS1/2 subcellular distribution was shown on the right. (n = 5 fields/coverslips, a representative from at least three experiments, Mean ± SEM, Student’s *t*-test or one-way ANOVA was used to determine the statistical significance for two groups or three groups comparison, respectively, * *p* < 0.05, ** *p* < 0.01, **** *p* < 0.0001). Scale bar, 20 µm.

Dsg3 plays a crucial role in junction assembly and has recently been identified as a potential component in the epithelial Hippo network [5,13,14]. To determine the coordinated regulation of Dsg3 with Hippo-YAP components in response to cell–cell contacts, further immunofluorescence was performed in N/TERT and T8 keratinocyte lines, seeded at various cell densities, i.e., sparse-30% density, intermediate-60% density and confluent-90% density. Cells were fixed and immunostained for Dsg3, p-YAP as well as LATS1/2 (which serves as the upstream kinase of YAP and is associated with the phosphorylation of YAP expression). Fluorescent microscopy showed mutual organization in protein expression and subcellular distribution accompanied by elevated cell densities. The expression of Dsg3 exhibited a steady increase coupled with the escalated cytoplasmic translocation of p-YAP/LATS1/2 in both N/TERTs (Figure 2A,B) and T8 cells (Figure 3A). The finding for p-YAP changes was consistent with our recent report based on oral keratinocytes [14]. Additionally, immunostaining for Dsg3, p-YAP and LATS1/2 in our generated stable T8 lines with hDsg3.myc transduction (D3) and the matched empty vector control (Vect Ct) [13] also showed the same trend, with elevated cytoplasmic signals of p-YAP and LATS1/2 in Dsg3 overexpressing cells in contrast to the Vect Ct line that showed predominantly nuclear signals of both p-YAP and LATS1/2 (Figure 3B). Moreover, enhanced p-YAP expression was detected in D3 cells by Western blotting analysis, similar to what we showed in oral keratinocytes [14] (data not shown). Furthermore, N/TERTs grown at various densities revealed a cell density-dependent increase in p-YAP, but not total YAP, along with Dsg3 (Figure 2C) and this trend was also observed in the time-course study for up to 8 days after calcium switching to induce junction formation (Figure 2D). Again, immunofluorescence agreed with the blot data and showed a bell-shaped expression profile for p-YAP and Dsg3 over time, i.e., when cells reached over confluence at day 8 the levels of both p-YAP and Dsg3 declined concurrently, suggesting their coordinate regulation in a time-dependent manner (image quantitation in Figure 2E, the representative images in Figure S1). Collectively, these findings are consistent with the notion that Dsg3 regulates YAP/p-YAP expression and cellular localization by recruiting both forms of proteins from the nucleus, the process we proposed potentially required for cell junction formation.

### 3.2. Treatment of Keratinocytes with Verteporfin Had Little Effect on Cell Viability

**Figure 4:**
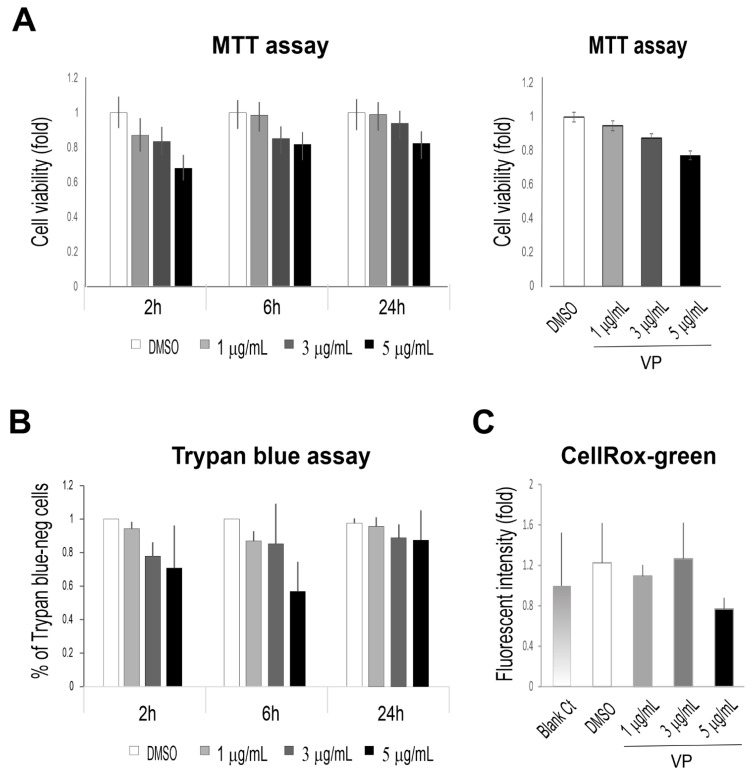
Treatment of N/TERT keratinocytes with VP shows little or no effect on cell viability. (**A**,**B**) N/TERT cells were treated with VP at various time frames and dosages as indicated in the figure. Cells were seeded in a 24-well plate in triplicate overnight before being treated with VP at increasing concentrations, i.e., 1, 3, and 5 µg/mL for 2, 6 and 24 h before MTT assay (**A**) and Trypan blue assay (**B**). (**C**) Measurement of the ROS levels in cells treated with VP. Cells was seeded in a 96-well plate overnight before being treated with VP at various concentrations for 6 h. Then, cells were incubated with CellRox reagent (5 µm) for 30 min before brief washing with PBS followed by image acquisition with an INCA 2200 Analyzer system straightaway. Image quantitation indicated no significant increase in ROS in cells treated with VP compared to controls (n = 25 automated fields/well, Mean ± SEM).

YAP phosphorylation at S127 results in its cytoplasmic localisation which is regarded as YAP inactivation. Our studies detected p-YAP colocalised with and sequestered by Dsg3 towards the cell surface and Dsg3 knockdown caused a defect in this process with enhanced p-YAP/YAP nuclear retention despite of general attenuation of the mRNA [13]. However, it remained unclear whether p-YAP was functionally required for cell–cell contact. Given our consistent finding that Dsg3 complexed with p-YAP, it was hypothesised that p-YAP might exert a cooperative function in Dsg3 mediated junction formation in the Hippo pathway. To address this question, we took the YAP inhibition approach by using verteporfin (VP), a well-characterised YAP inhibitor [8,9,16], and asked whether VP had an impact on cell junction formation and adhesive functionality. VP has recently been identified as a potential anti-tumour therapeutic drug by targeting YAP and inducing apoptosis while suppressing cell survival [8,16]. To determine VP treatment had any cytotoxic effect, first, we analysed cell viability in cells exposed VP by the MTT and trypan blue assays, as well as the measurement of reactive oxygen species (ROS), in N/TERT cells, treated with VP at increasing concentrations, i.e., 1, 3 and 5 µg/mL, the concentrations used by others [8,9,26], for various time frames up to 24 h. Cells were seeded at approximately 70~80% confluence overnight before being treated with VP before cytotoxicity assays to assess the cell viabilities (Figure 4A–C). In general, the results showed no statistical significance in these experiments although a trend of decline in cell viability was observed in cells exposed to the high dosages of VP by MTT and trypan blue assays. Together these findings suggest that treatment of VP at concentrations of up to 5 µg/mL for 24 h had only a merely subtle cytotoxic effect in N/TERT cells in this study.

### 3.3. Verteporfin Causes a Drastic Reduction in YAP and p-YAP with Concomitant Loss of the Majority of Desmosomal Genes and Proteins

**Figure 5:**
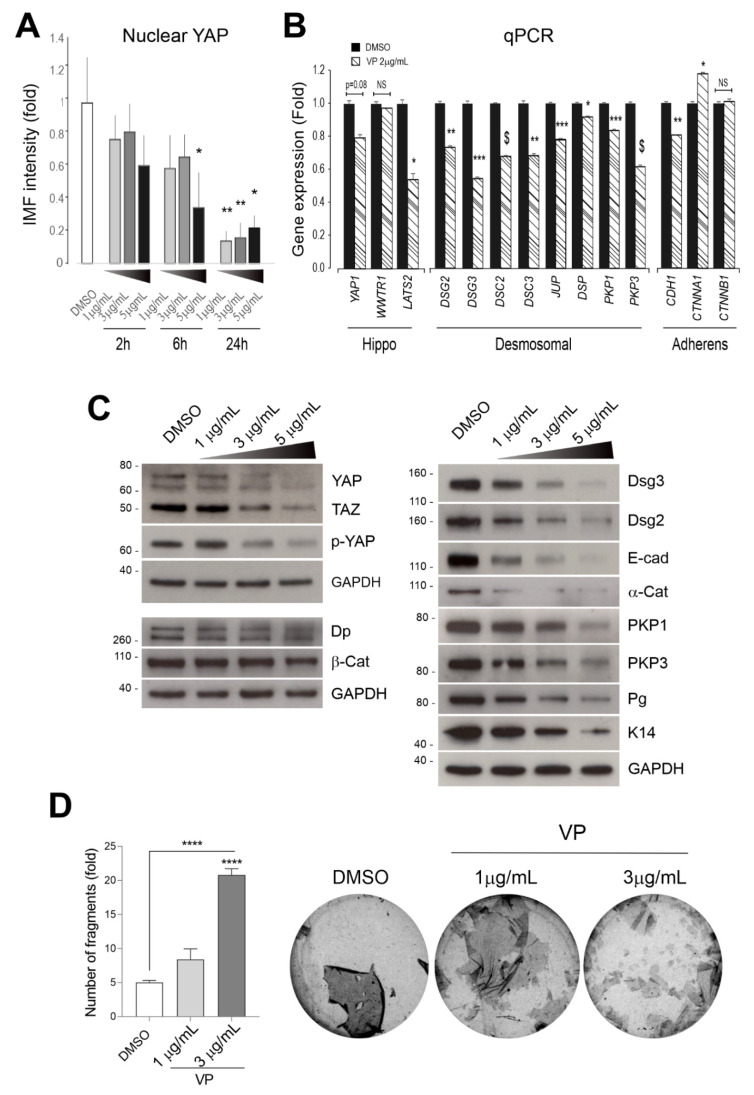
Desmosomal genes are the targets of YAP nuclear transcription activity and VP treatment causes drastic suppression of cell junctional proteins in N/TERT cells. (**A**) IMF analysis of YAP nuclear expression in cells treated with VP at different concentrations for various time points indicated a time and a dose-dependent reduction in nuclear YAP. (**B**) qPCR analysis for various cell anchoring junctional genes in N/TERTs treated in the presence and absence of VP at 2 µg/mL for 6 h (n = 4, Student’s *t*-test was used to determine the *p* values, * *p* < 0.05, ** *p* < 0.01, *** *p* < 0.001 and $ *p* < 0.0001). NS, no significance. Note that the *p*-value for *YAP1* was *p* = 0.08 based on the current test. (**C**) Western blotting analysis for the indicated cell junction assembly proteins showed a remarkable decrease, except for Dp and β-Catenin, in cells with treatment of VP in a dose-dependent manner, compared to the respective controls. Cells were treated with VP at various concentrations for 6 h before protein extraction. Dp: desmoplakin; E-Cad: E-cadherin; PKP1: plakophilin 1; PKP3: plakophilins 3; β-Cat: β-Catenin. (**D**) Dispase cell dissociation assay showed compromised cell–cell adhesion strength in cells treated with VP in a dose-dependent manner. N/TERT cells were seeded at confluent densities and grown for three days to allow the junctions to become established. Then, cells were treated with VP for the indicated concentrations alongside vehicle control for 6 h before dispase treatment at a 2.4 unit/mL concentration until the epithelial cell sheets detached from the substrate. This was followed by mechanical stress to induce fragmentation as displayed in the images on the right, and the quantitation of fragments in cells treated in the presence and absence of VP is shown on the left (Mean ± SEM, one-way ANOVA was used to determine the statistical significance, **** *p* < 0.0001).

Our recent studies have shown that YAP depletion resulted in elevated expression of cell junction assembly proteins, including Dsg3 and α-Catenin [5,14]. We expected a similar finding in cells with YAP inhibition such as VP treatment. To test this possibility, we added VP in the culture medium of N/TERT cells at increasing concentrations and incubated cells for various periods up to 24 h. YAP nuclear expression was firstly measured by immunofluorescence and image quantitation indicated a dose and a time-dependent decrease in nuclear YAP in cells treated with VP (Figure 5A). Then, qPCR analysis was performed for various junctional genes, however, to our surprise, we detected a significant reduction in the majority of anchoring junctional genes, including eight desmosomal genes plus adherens junctional components *CDH1* (which encodes for E-cadherin), except for *CTNNA1* (which encodes for α-Cat) that showed an increase and *CTNNB1* (which encodes for β-Cat) that showed no change compared to the respective controls (Figure 5B). In addition, *LATS2* also showed a reduction in VP treated cells. However, both *YAP1* and TAZ (*WWTR1*) had no significant changes between control and VP treated cells. The Western blotting analysis supported the gene expression data except for YAP/p-YAP, as well as TAZ, which exhibited evident reduction at the protein levels, following VP treatment. A remarkable dose-dependent suppression was observed in both desmosomal and adherens junctional components as well as keratin 14 (K14) in N/TERTs treated with VP except for Desmoplakin (Dp) and β-Cat that displayed relatively fewer changes (Figure 5C). In addition, the functionality of cell–cell adhesion strength in VP treated cells was assessed by the dispase cell dissociation assay as described previously [5,28]. Cells were seeded at confluent densities and grown for a couple of days to allow the junctions to become established. Then, cells were subjected to VP treatment at two different dosages, i.e., 1 µg/mL and 3 µg/mL, for 3 h before the dispase assay. The detached epithelial sheets of different conditions were subjected to equal mechanical stress to induce fragmentation (Figure 5D). Quantification of fragments showed a clear compromised cell cohesion induced by VP in a dose-dependent manner. Collectively, these results confirmed that VP treatment causes the weakening of cell–cell adhesion due to the dissolution of adhesion proteins and disintegration of cell junctions with concurrent loss of Dsg3 and p-YAP at the plasma membrane of keratinocytes. Furthermore, the data from the qPCR and Western blotting analyses indicate that all the desmosomal genes analysed as well as E-cadherin are the targets of the YAP-TEAD transcription activity since their inhibition by VP attenuated both the gene and protein expression of these junctional components.

### 3.4. Overexpression of Dsg3 Can Rescue, at Least in Part, the VP-Induced Impairment to Cell Junctions

**Figure 6:**
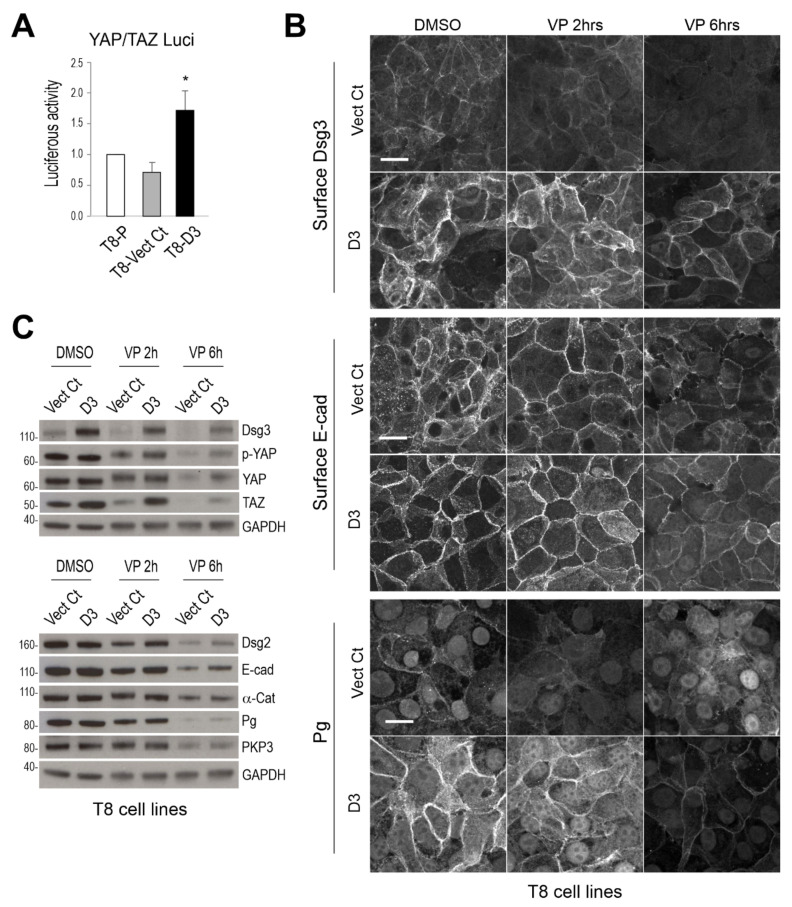
Overexpression of Dsg3 can compensate VP-induced attenuation of junction protein expression and disruption of the anchoring junction architecture in T8 keratinocytes. (**A**) YAP/TAZ luciferase assay in skin-derived T8 carcinoma cell lines with transduction of hDsg3.myc (T8-D3) and matched vector control (Vect Ct) alongside parental cells (T8-P). Cells were transfected with the YAP/TAZ plasmid (8xGTIIC) for 24 h before the luciferase assay. Relatively higher luciferase activity of YAP/TAZ was detected in the T8-D3 cell line compared to controls (representative of three independent experiments, * *p* < 0.05). (**B**) Confocal images in Vect Ct and D3 cells that were treated with VP at 3 µg/mL for 2 and 6 h before formaldehyde fixation only without Triton and then immunostained for surface Dsg3 (5H10) and E-cadherin (HECD-1), both of which bind to the N-terminus of the extracellular domains of cadherins, as well as plakoglobin (Pg) in cells after being treated with Triton. Compensation for junction formation in both the E-cadherin and Pg staining was shown in D3 cells with overexpression of Dsg3 compared to Vect Ct cells treated with VP. Note that pronounced nuclear Pg was detected in Vect Ct with marked suppression in D3 cells (images were representative of three independent experiments). (**C**) Western blotting analysis for various cell junction assembly proteins in T8 Vect Ct and D3 lines exposed to VP at 3 µg/mL for 2 and 6 h. Again, compensation was detected for various proteins in D3 cells treated with VP. Scale bar, 20 µm.

To determine the specific role of Dsg3 in VP-induced junctional changes, next, we asked whether Dsg3 overexpression could rescue the disruption of cell–cell junctions. To this end, we took advantage of our generated stable T8-D3 and Vect Ct lines. First, we assessed the activity of YAP in both cell lines by the luciferase assay. Cells were transfected with an equal amount of the YAP/TAZ plasmid (8xGTIIC) [27] for one day before the assay. The result indicated enhanced YAP/TAZ transcription activity in D3 cells compared to Vect Ct or parental control cells (Figure 6A), suggesting Dsg3 overexpression promoted YAP/TAZ transcriptional activity. Next, we analysed the surface expression of Dsg3 and E-cadherin (without Triton treatment) as well as plakoglobin (Pg) (with Triton permeabilization), the Armadillo protein located in the cytoplasmic plaque of desmosomes and has a dual role in both the junction and nucleus, in both Vect Ct and D3 lines treated with 3 µg/mL VP for 2 and 6 h, respectively. Cells treated with DMSO served as vehicle control. Here, both Dsg3 and E-cadherin were labelled with anti-Dsg3 (5H10) and E-cadherin (HECD-1) antibodies that bind to the N-terminus of the extracellular domain on the cell surface. Confocal microscopy revealed enhanced Dsg3 expression in D3 cells as expected. In line with our previous finding that overexpression of Dsg3 enhances surface E-cadherin expression [13], D3 cells with hDsg3.myc transduction also showed well-organised or enhanced membrane staining of E-cadherin and Pg in both vehicle controls and VP-treated cells although a time-dependent decline in fluorescent intensities was noticeable (Figure 6B). In contrast, Vect Ct cells in each treatment exhibited relatively weaker signals for these proteins with disorganised membrane structures. In addition, the pronounced Pg nuclear signals were observed in both the control and VP treated Vect Ct cells, indicating disruption of cell junctions leading to nuclear translocation of Pg. Overexpression of Dsg3 recused this phenotype and sequestered Pg to the plasma membrane with concomitant reduction in its nuclear localisation. Since T8 is a carcinoma cell line, it is not surprising that the architecture of cell junctions was disorganised in Vect Ct cells. However, Dsg3 overexpression helped restore the proper junction formation and architecture in these cells (Figure 6B) demonstrating a vital role of Dsg3 in governing cell junction formation.

**Figure 7:**
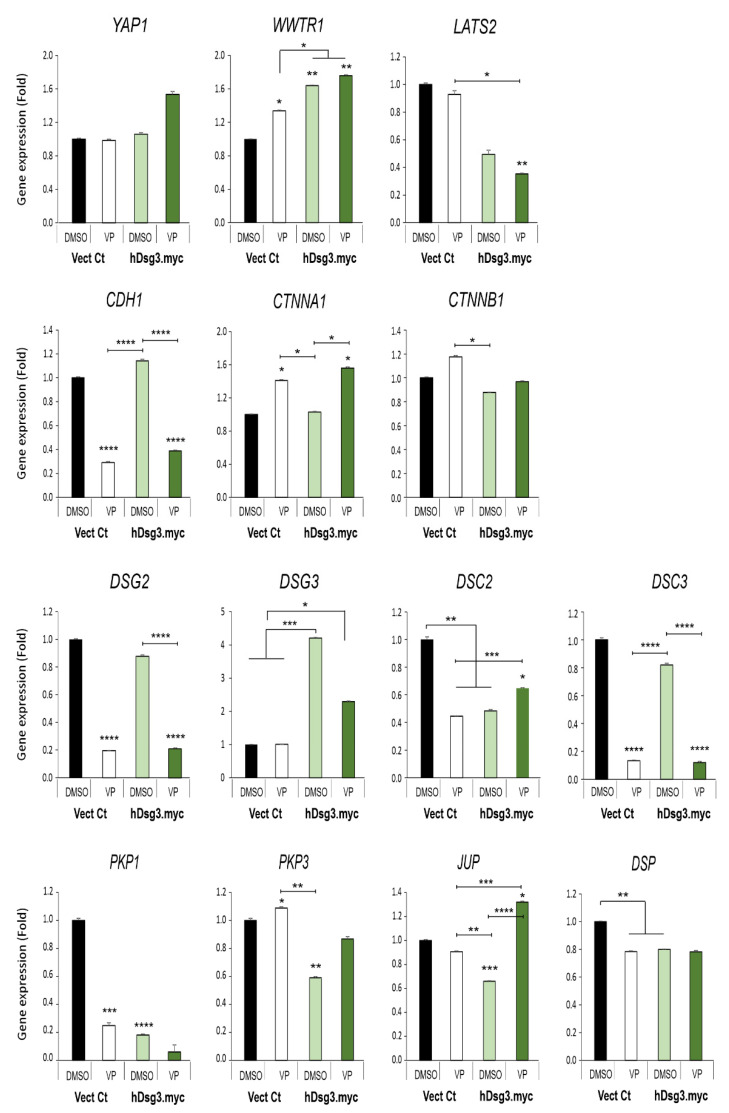
Effect of Dsg3 overexpression and VP treatment on the expression of various Hippo and junctional genes in T8 keratinocyte cell lines. qPCR data showed variations of individual genes in cells with Dsg3 overexpression and treated in the presence and absence of VP at 3 µg/mL for 6 h. Increased expression of *WWTR1* but a decrease in *DSC2*, *PKP1/3*, *JUP* and *DSP* were detected in D3 cells compared to the Vect Ct line. Increased expression of *WWTR1* and *CTNNA1* was also shown to be induced by VP treatment, but only *DSC2* and *JUP* displayed compensation in D3 cells with overexpression of Dsg3 relative to controls without VP exposure (n = 4, error bar: Mean ± SEM, * *p* < 0.05, ** *p* < 0.01, *** *p* < 0.001, **** *p* < 0.0001).

In parallel, the VP treated cells (at 3 µg/mL for 2 and 6 h) were also analysed by Western blotting. Despite a general trend of decline in all proteins following VP treatment, overexpression of Dsg3 in D3 cells showed compensation or rescue for the loss of junctional proteins as compared with the respected controls, such as YAP/p-YAP, TAZ, Dsg2, E-cadherin, especially at 6 hours’ time point (Figure 6C). Notably, increased levels in YAP and p-YAP, as well as TAZ, were evident in D3 cells compared to Vect Ct in each condition. These findings were consistent with the notion that Dsg3 regulates YAP by inducing p-YAP expression and stabilising the adhesion proteins required for junction formation. Furthermore, the qPCR analysis for various cell junctional genes in both cell lines treated with VP at 3 µg/mL for 6 h (Figure 7) indicated that several genes such as *LATS2*, *DSC2*, *PKP1/3, JUP* and *DSP* were negatively regulated by Dsg3. Consistent with Western blot data, *WWTR1* showed to be positively regulated by Dsg3, with a nearly 2-fold increase in D3 cells relative to Vect Ct (Figure 7). Treatment of Vect Ct cells with VP evoked an increase in *WWTR1*. Although no statistical significance was found for *YAP1* between different conditions, there was a trend of increase in D3 with VP treatment. Notably, all five cadherin genes analysed showed a remarkable suppression by VP treatment, the results consistent with that of N/TERTs (Figure 5B). However, among five cadherin genes, only *DSC2* exhibited compensation upon VP treatment in D3 cells. Both *CTNNA1* and *CTNNB1* also showed similar results to N/TERTs, with elevated expression in *CTNNA1* indicating mutual exclusive regulation between *CTNNA1* and *YAP1* as shown in our previous studies [5,14]. Apart from *PKP1* and *DSP* with the expression being suppressed by VP, *PKP3* and *JUP* displayed different responses from that of N/TERT cells, especially *JUP* that also showed compensation, such as *DSC2*, in the D3 cells treated with VP. Collectively, these data suggest cell context-dependent variations between normal and carcinoma cell lines in the Hippo pathway. Thus at the gene levels, at least *DSC2* and *JUP* exhibited compensation upon VP exposure in Dsg3 overexpressing cells.

Taken together, this study suggests a vital role of Dsg3 in fine-tuning the Hippo components in order to maintain the balance of the Hippo network. The results from these gain-of-function study approaches in T8 cells agreed with the findings obtained in N/TERTs and suggest that the desmosomal genes plus E-cadherin are regulated by YAP activity and overexpression of Dsg3 can, at least in part, rescue the VP-induced defect in cell–cell adhesion and junctional integrity via augmented stability of p-YAP upon VP exposure.

### 3.5. Dsg3 and p-YAP Show Coordinated Expression in Response to VP Treatment

**Figure 8:**
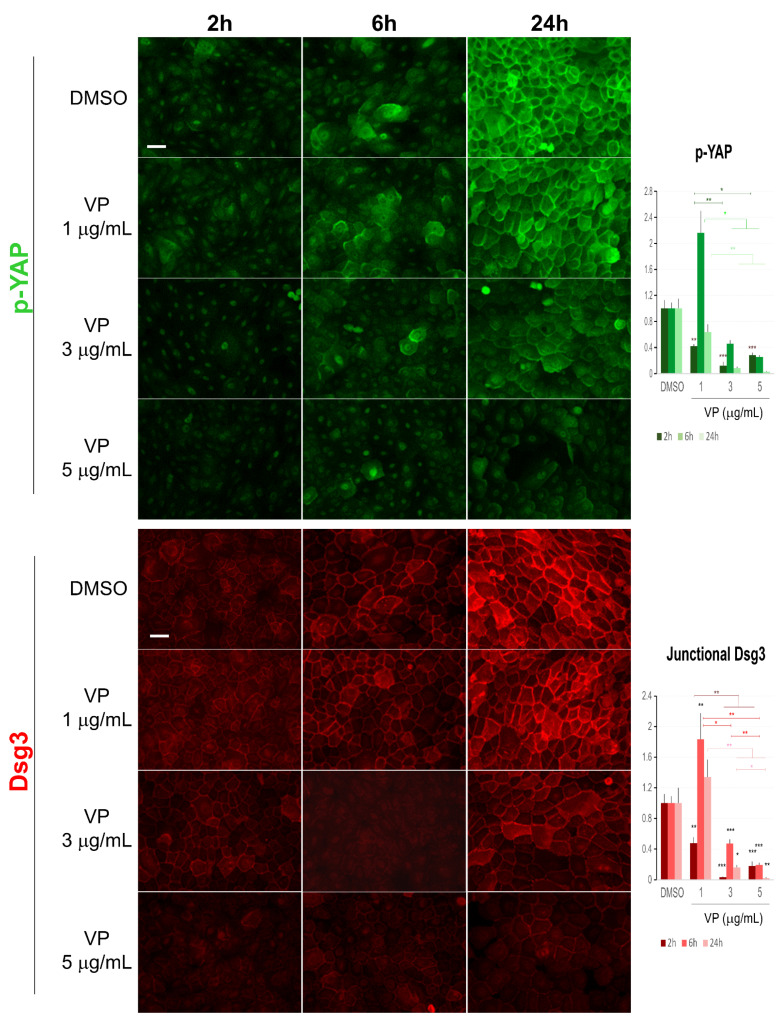
Immunofluorescence for Dsg3 and p-YAP indicates their concurrent regulation in N/TERT cells in response to VP treatment. The epi-fluorescent microscopic images showed a clear trend of a dose and time-dependent reduction in Dsg3 and p-YAP in cells treated with VP at increasing concentrations, i.e., 1, 3, and 5 µg/mL for 2, 6 and 24 h. Image quantitation for both proteins was shown in the bar charts on the right. IMF intensities for each time point were normalised against DMSO vehicle control (n = 5 images/coverslips, data were the representative of at least three independent experiments, Mean ± SEM, two-way ANOVA was used to determine the statistical significance, * *p* < 0.05, ** *p* < 0.01, *** *p* < 0.001). Note that a concurrent sharp increase in both Dsg3 and p-YAP was shown in cells exposed to VP at the concentration of 1 µg/mL for 6 h, but in general, there was a trend of a time and dose-dependent loss of both proteins in the VP treated cells compared to DMSO controls. Scale bar, 20 µm.

**Figure 9:**
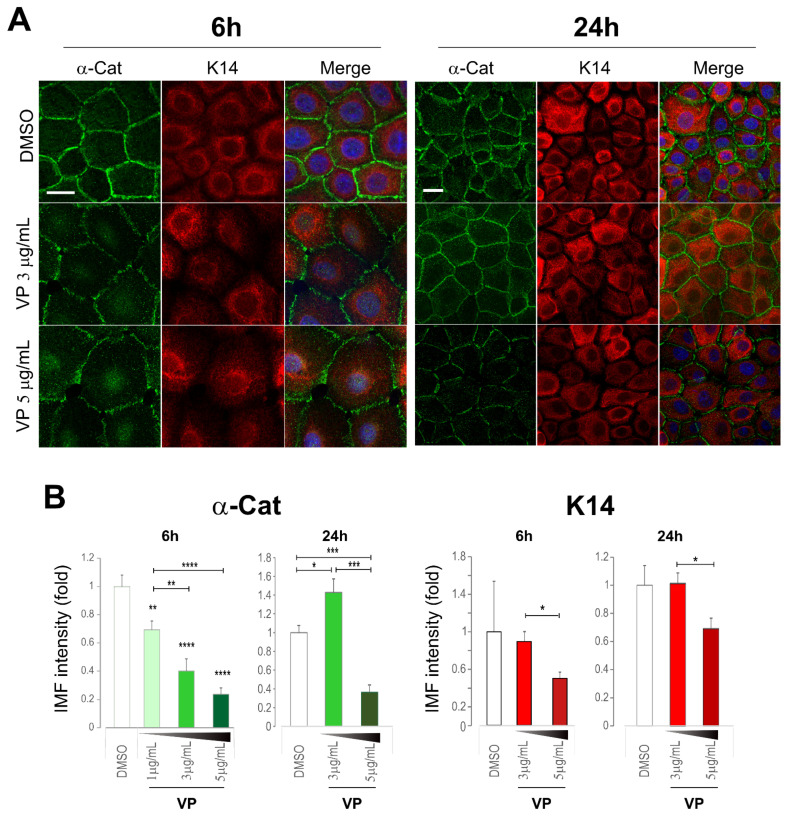
Immunofluorescent analysis of α-Catenin and keratin 14 also showed a dose-dependent reduction in VP treated cells. (**A**) Confocal images of N/TERTs were treated in the presence and absence of VP for 6 and 24 h, respectively, and double labelled for α-Catenin (α-Cat) and keratin 14 (K14). K14 perinuclear retraction was apparent in VP treated cells at 6 h time point compared to DMSO control. (**B**) Image quantitation for the protein staining shown in **A** (n = 5 images/coverslips, data were a representative of two independent experiments, Mean ± SEM, one-way ANOVA was used to determine statistical significance, * *p* < 0.05, ** *p* < 0.01, *** *p* < 0.001, **** *p* < 0.0001). A clear dose-dependent reduction in both proteins was shown in VP-treated cells at 6 h time point. At the 24 h, however, an increased expression of α-Catenin was detected in the VP treated cells at the concentration of 3 µg/mL but with a marked reduction at the concentration of 5 µg/mL accompanied by decreased K14. Scale bar, 20 µm.

Finally, immunofluorescent staining for p-YAP along with various junctional proteins, including both the desmosomal and adherens junction proteins, was performed in VP treated cells. It was reported that VP induces elevated expression and cytoplasmic translocation of p-YAP [9]. However, here we showed that double-staining for Dsg3 and p-YAP revealed a clear dose and time-dependent suppression of both proteins in cells exposed to VP in N/TERT keratinocytes, especially the loss at the multicellular junctions by confocal microscopy (Figure 8 and Figure S2). Notably, the peaks with a sharp increase in both Dsg3 and p-YAP were observed in cells treated with VP at 1 µg/mL for 6 h compared to the respective controls, suggesting their coordinated regulation with elevated expression in response to VP treatment at such a lower concentration. In addition to a trend of p-YAP nuclear retention coupled with the loss of its membrane distribution was seen in cells exposed to VP in a dose-dependent manner, indicating perturbation in the process of Dsg3 mediated p-YAP cytoplasmic translocation due to Dsg3 degradation induced by VP (Figure 8). Similar results with decreased expression were also detected for E-cadherin, α-Catenin and K14 staining in the VP treated cells in a dose-dependent manner (Figure 9 and Figure S3). α-Catenin also exhibited a small increase in cells treated with VP at 3 µg/mL for 24 h. Enhanced cell size coupled with K14 cytoplasmic retraction was shown in VP treated cells compared to control, though the fluorescent intensities were not severely affected by VP treatment at 3 µg/mL (Figure 9). Interestingly, Dp showed an opposite effect with a significant increase in cells treated with VP at both the 1 µg/mL and 3 µg/mL concentrations (Figure S4).

Taken together, these results suggest that VP has a detrimental impact on protein stability of YAP/p-YAP as well as the majority of cell junction assembly proteins, leading to junction disintegration and failure of cell–cell adhesion. Additionally, these findings also support further the notion that the complex of Dsg3/p-YAP may be required in keratinocyte junction formation and therefore imply a biological function of p-YAP in this process, rather than a redundant protein present in the cytoplasm of cells.

## 4. Discussion

Intercellular junctions are key factors in controlling the activity of the Hippo pathway, cellular polarity and tissue morphogenesis, whereas their deregulation contributes to the development of a variety of diseases, including cancer and blister-associated skin fragilities. Although both tight junctions and adherens junctions have been identified to influence the normal activity of the Hippo pathway, little is known about desmosomes and their constitutive components in this context [29]. Our recent studies have shown that Dsg3 forms a complex with p-YAP-S127 and recruits it to the cell surface [13,14]. This study further consolidated this finding in normal keratinocytes. In addition, we investigated the potential role of the Dsg3/p-YAP complex in keratinocyte junction formation which could have implications for epithelial cell development and regeneration. We showed that disruption of YAP interaction with TEAD by VP caused remarkable suppression of desmosomal cadherins, including Dsg3 that led to a reduction in YAP and p-YAP with concomitant attenuation of cell junction formation and integrity. Overexpression of Dsg3 was able to rescue, at least in part, such defects with enhanced surface expression and architecture of E-cadherin and Pg as well as the stability of other junction assembly proteins. Importantly, a significant downregulation of the desmosomal genes coupled with a decrease in desmosomal proteins was detected in cells treated with VP in a time and dose-dependent manner, indicating a likelihood for the desmosomal genes serving as the targets of the Hippo-YAP pathway. Hence, YAP activation can trigger the desmosomal gene expression and the complex formation of Dsg3/PKPs/p-YAP/14-3-3 to facilitate junction configuration and epithelial regeneration [13,30,31]. Furthermore, we showed that Dsg3 and p-YAP exhibited coordinated expression and cellular distribution in response to cell densities and the duration of cell cultures. Thus the findings from this study revealed previously unrecognised functions of the Hippo-YAP pathway and suggest that (1) desmosomal components are likely the targets of YAP-TEAD transcription activity and (2) p-YAP may serve as a Hippo component engaging in Dsg3 mediated epithelial cell junction construction in keratinocytes.

**Figure 10:**
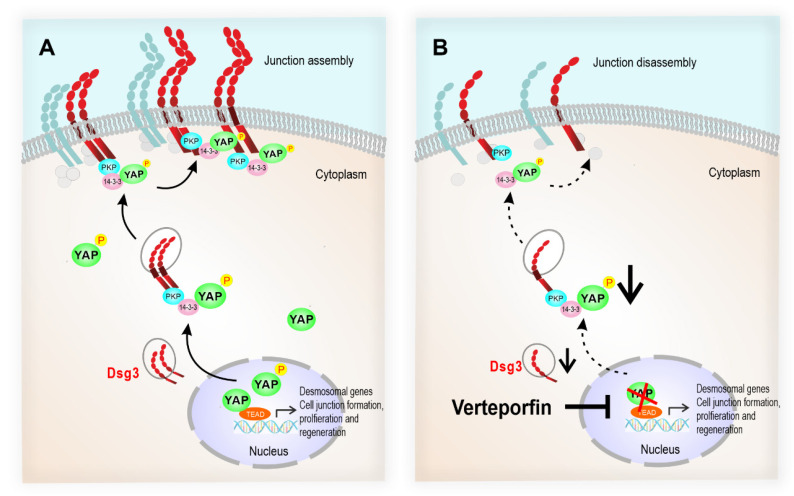
The schematic model illustrates that the desmosomal genes are the YAP transcriptional targets, and the Dsg3/p-YAP complex is required for epithelial cell junction formation. (**A**) Upon activation of YAP during the epithelial regeneration, the expression of Dsg3, along with other cell junction proteins, is induced which leads to YAP phosphorylation and cytoplasmic translocation and eventually sequestered by Dsg3 to the cell surface to facilitate junction assembly. (**B**) In contrast, YAP inhibition by Verteporfin causes suppression of desmosomal gene transcription including Dsg3 and therefore the attenuation of the Dsg3/p-YAP complex, leading to the defect in junction assembly and ultimately the disintegration of cell junctions.

YAP shuttles between the cytoplasm and nucleus and this process is regulated by phosphorylation through its upstream Hippo kinases LATS1/2, leading to its cytoplasmic translocation and retention via binding to 14-3-3 [2,7]. Thus, p-YAP is regarded as a redundant protein in the Hippo signalling pathway. Our recent reports have identified a protein complex containing Dsg3/p-YAP at the cell borders in both the oral and skin keratinocytes [13,14], but its function remained obscure. The activity of the Hippo-YAP pathway is known to be varied depending on the cell context. A previous study reported that cytoplasmic YAP is required for endothelial cell migration and angiogenesis via the mechanism of regulating the activity of the small GTPase Cdc42 [32]. However, our recent study based on oral keratinocyte cell lines indicated that the complex formation of Dsg3/p-YAP prevents collective cell migration via inhibiting the EGFR-Hsp27-YAP axis [14]. Moreover, transient transfection of human Dsg3 plasmid in HaCaT keratinocytes [25] resulted in a gene dosage-dependent expression of surface E-cadherin along with Dsg3/p-YAP, and Dsg3 depletion rendered an inverse effect with a reduction and pronounced nuclear retention of p-YAP coupled with junction disruption [13]. Here, we confirmed further the existence of a complex of Dsg3/p-YAP in normal N/TERT keratinocytes and showed their coordinated expression and subcellular distribution in various culture conditions as well as in response to VP treatment. Dsg3 plays an essential role in junction formation and cell–cell adhesions. Treating cells with VP caused severe suppression of Dsg3 and YAP/p-YAP among other adhesion proteins of anchoring junctions, including desmosomes and adherens junction, and disintegration of cell junction structures. Therefore, our data suggest that p-YAP may be required in the Dsg3 mediated junction assembly. Epithelial junction formation is a dynamic intricate process that requires a special and temporal orchestrated regulation of several signalling pathways in conjunction with the assembly of adhesion proteins at the plasma membrane. The protein complex containing Dsg3/PKPs/14-3-3 has been identified to be required for desmosome assembly [13,30,31]. Our studies provided evidence that p-YAP served as a constitutive molecule in such a complex and YAP inhibition led to severe perturbation of such a complex and thus junction disassembly as illustrated in Figure 10. Hence, we argue that p-YAP not only physically interacts with this protein complex but may also actively participate in the process of epithelial regeneration [13]. Thus, p-YAP is not a redundant protein but rather is engaging in Dsg3 mediated desmosome assembly in keratinocytes. Additionally, this model also suggests that YAP transcriptional activity is required for desmosomal gene expression essential for keratinocyte adhesion and morphogenesis [7]. However, the binding between Dsg3 and p-YAP is likely to be indirect via PKPs/14-3-3 [30,31], as shown in our previous study that PKP1 is required for p-YAP stability and junction assembly [13]. It is also worth pointing out that these results differ completely from the finding of RNAi-mediated knockdown of YAP that caused enhanced expression of Dsg3 and α-Catenin [5,14], the result likely caused by compensation of TAZ (Figure 1C, Figure 6C and Figure 7) that binds to TEAD to mediate transcription of the same set of target junctional genes.

Increasing evidence indicates that VP, a photosensitizer, can be used as a potential therapeutic drug in the treatment of an array of cancers that blocks the expression of YAP target genes accompanied by suppression of cell proliferation [19,21,22,33]. However, relatively little is known about the impact of VP on cell junctions except for limited reports about its influence on tight junctions and adherens junctions [33,34,35]. An earlier study showed that application of VP at a therapeutic concentration in conjunction with a non-thermal laser causes morphologically and functionally detectable breakdown of the outer blood-retinal barrier function without any damage to retinal pigment epithelial cells, but with increasing dosage resulted in cell damage [34]. These findings imply that VP mediated YAP inhibition can impair the intercellular junction with a therapeutic concentration without causing cell damage. Another study showed that YAP is required in the early blastocyst development to facilitate transcriptional regulation of the genes essential for lineage commitment, including proper tight junction formation [35]. Decreased YAP activity is also associated with lung injury and adherens junction disassembly, whereas increased YAP activity facilitated lung repair with enhanced adherens junction reassembly [36]. Collectively, these findings underscore that YAP at the physiological expression levels is required for epithelial junction formation, cell cohesion and morphogenesis. Indeed, in this present study, we demonstrated that the YAP-TEAD activity impacts the expression of desmosomal genes, including Dsg3 which is required for sequestering the junction assembly complex containing Dsg3/p-YAP to the cell surface and facilitates junction formation. In contrast, YAP inhibition impaired this entire process, resulting in junction disintegration. A coordinated temporal increase in both Dsg3 and p-YAP was observed in cells treated with VP at 1 µg/mL for 6 h with a similar finding observed with α-Catenin and Dp, suggesting stimulation of Hippo signalling by VP. As reported in the literature [9], the inhibition of YAP could lead to its enhanced phosphorylation and cytoplasmic accumulation due to Hippo activation. Our recent report has identified several desmosomal genes that were functionally associated with *YAP1* [14] and the findings in this study indicated that Dsg3 plays a crucial role in the Hippo network to fine-tune the expression and balance of Hippo components. This notion was supported by our qPCR data that *LATS2*, *DSC2*, *PKP1/3*, *JUP* and *DSP* were negatively regulated, and *WWTR1*/TAZ was positively regulated by Dsg3 in T8 cells; the result also agreed with the luciferase assay, and that overexpression of Dsg3 could compensate for the VP-induced suppression of *DSC2* and *JUP* in these cell lines. In addition, protein analyses showed that overexpression of Dsg3 could compensate, at least to some extent, for VP-induced dissolution of E-cadherin/Pg and disability of other adhesion proteins. It is worth noting that the dosages of VP used in this study had little or no effect on cell viability since no statistical significance was found in the MTT and trypan blue assays, as well as the measurement of the ROS levels in cells exposed to VP at various concentrations and for different time frames, compared to the respective controls.

In conclusion, this study provided the first evidence that the desmosomal genes are likely the YAP-TEAD transcriptional targets and demonstrated that Dsg3 regulates YAP expression via forming a complex with and recruits p-YAP from the nucleus towards the cell surface necessary for cell junction assembly and epithelial regeneration. Inhibition of YAP-TEAD interaction by VP treatment severely impaired this process, leading to defects in desmosome assembly and attenuation of keratinocyte cell–cell adhesion.

## Supplementary Materials

The following supporting information can be downloaded at: https://www.mdpi.com/article/10.3390/life12060792/s1, Figure S1: Confocal microscopy of N/TERT cells double-stained for Dsg3 and p-YAP in a time-course experiment. Figure S2: Confocal images of immunofluorescent staining for Dsg3 and p-YAP in N/TERT cells treated with VP. Figure S3. Immunofluorescence for E-cadherin in N/TERT cells treated with VP. Figure S4. Immunofluorescence for Desmoplakin in N/TERT cells treated with VP.
