# Supplementary material for: YAP Inhibition by Verteporfin Causes Downregulation of Desmosomal Genes and Proteins Leading to the Disintegration of Intercellular Junctions
Source: Life (Basel). 2022 May 26;12(6):792. doi: 10.3390/life12060792 (PMC9225343; doi:10.3390/life12060792)
Supplement: Supplementary file 1 [file life-12-00792-s001.zip › life-1674887-supplementary.pdf]

## Supplemental Materials

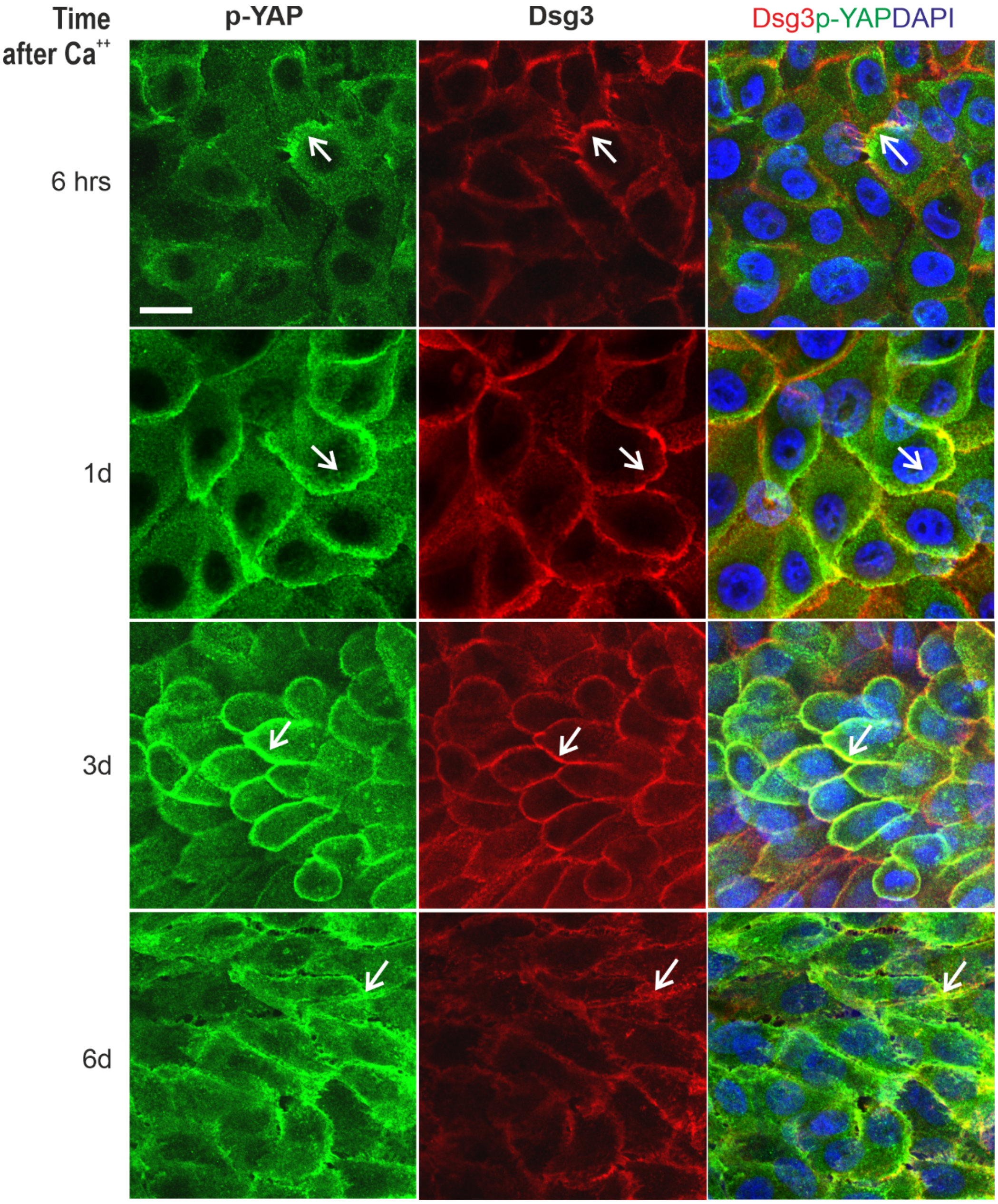


**Figure S1.** Confocal microscopy of N/TERT cells double-stained for Dsg3 and p-YAP in a time-course experiment. Cells were seeded at confluent densities in KSFM at low calcium (0.09mM) before being replaced with KGM containing normal calcium concentration (1.8mM) and were allowed to grow for various time frames before fixation followed by immunostaining. The representative images were displayed here with the corresponding image quantitation being shown in Figure 2E. Arrows indicated co-localisation of the two proteins at the plasma membrane. A gradual corresponding increase of both proteins was seen till day 3 before a decline on day 6. Scale bar, 10 µm.

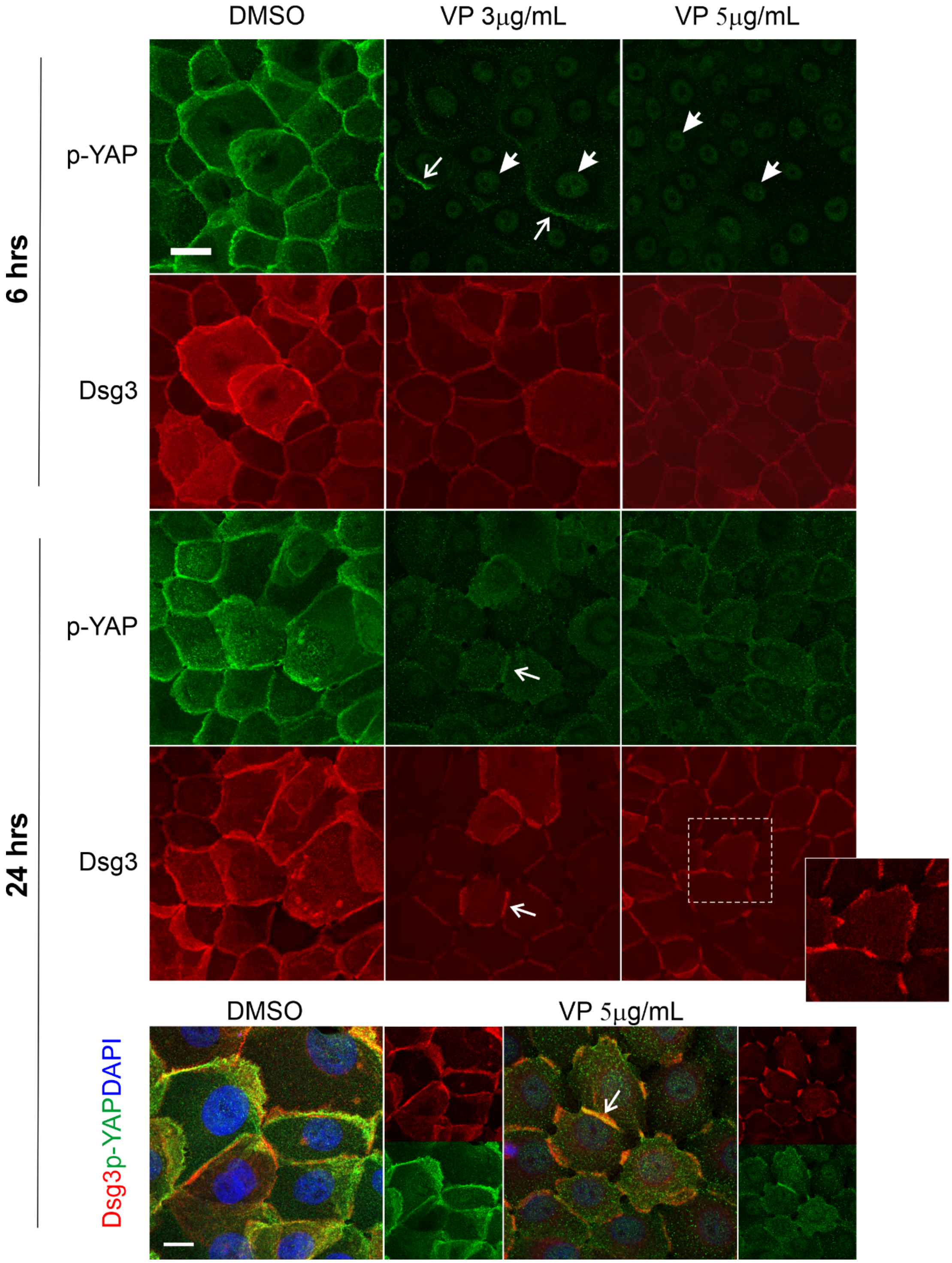


**Figure S2.** Confocal images of immunofluorescent staining for Dsg3 and p-YAP in N/TERT cells treated with VP. The representative confocal images showed a drastic effect of VP on the protein stability of Dsg3 and p-YAP. Cells were treated with VP at increasing concentrations, i.e. 3, and 5 µg/mL for 6 and 24 hours, respectively. Note a clear dose and a time-dependent reduction of both proteins were shown in cells exposed to VP, especially at multi-cellular junctions as pointed by the arrows as well as the insert. Arrowheads indicate the p-YAP nuclear signals in VP treated cells. Scale bar, 10 µm.

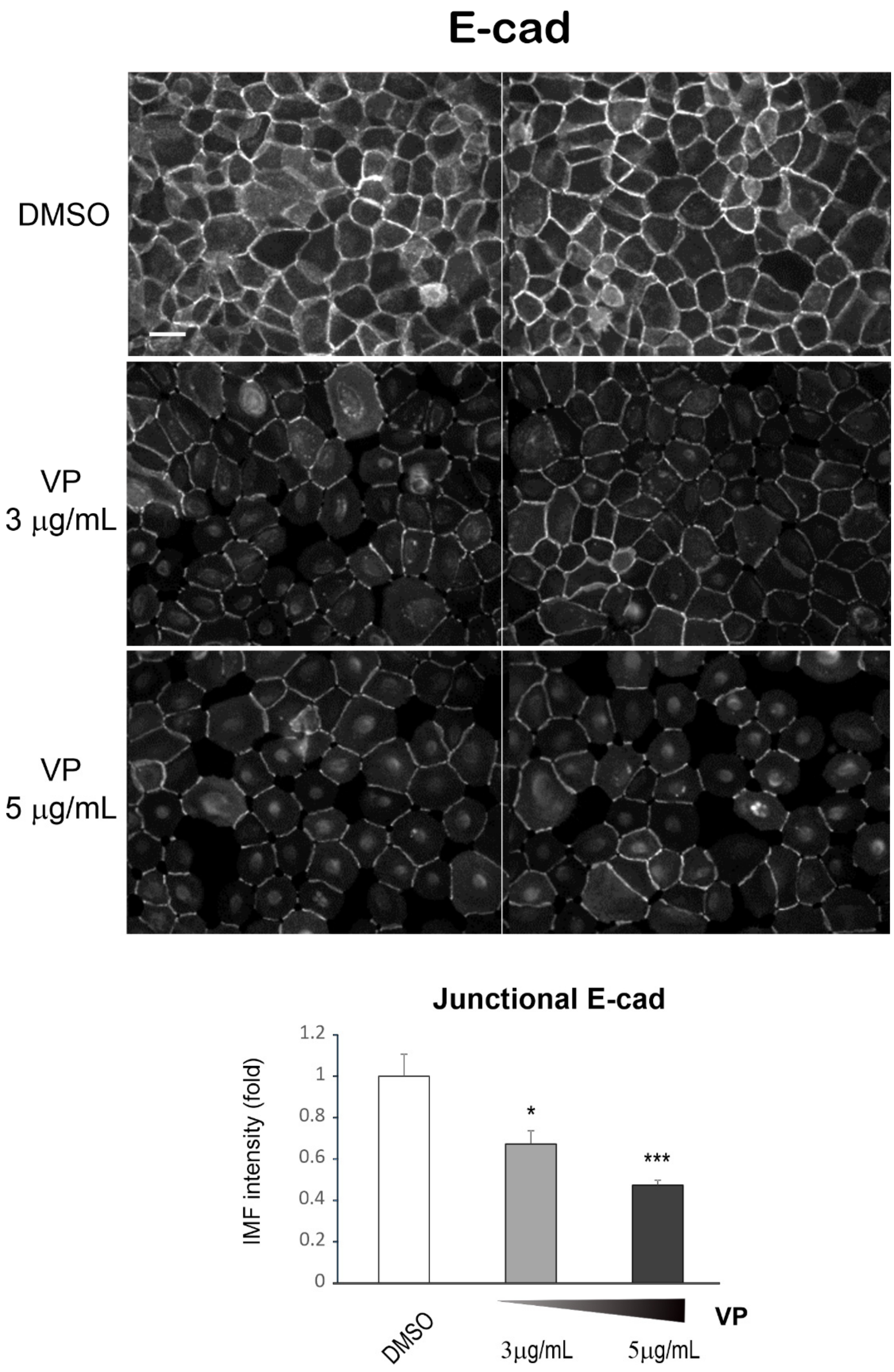


**Figure S3**. Immunofluorescence for E-cadherin in N/TERT cells treated with VP. The epi-fluorescent microscopic images showed a dose-dependent reduction of E-cadherin (E-cad) in cells treated with VP at 3 and 5 μg/mL concentrations, respectively, alongside DMSO vehicle control. Image quantitation was shown underneath (n=5 fields/coverslips, Mean ± SEM, one-way ANOVA was used to determine the p values, *p<0.05, ***p<0.001). Scale bar, 20 μm.

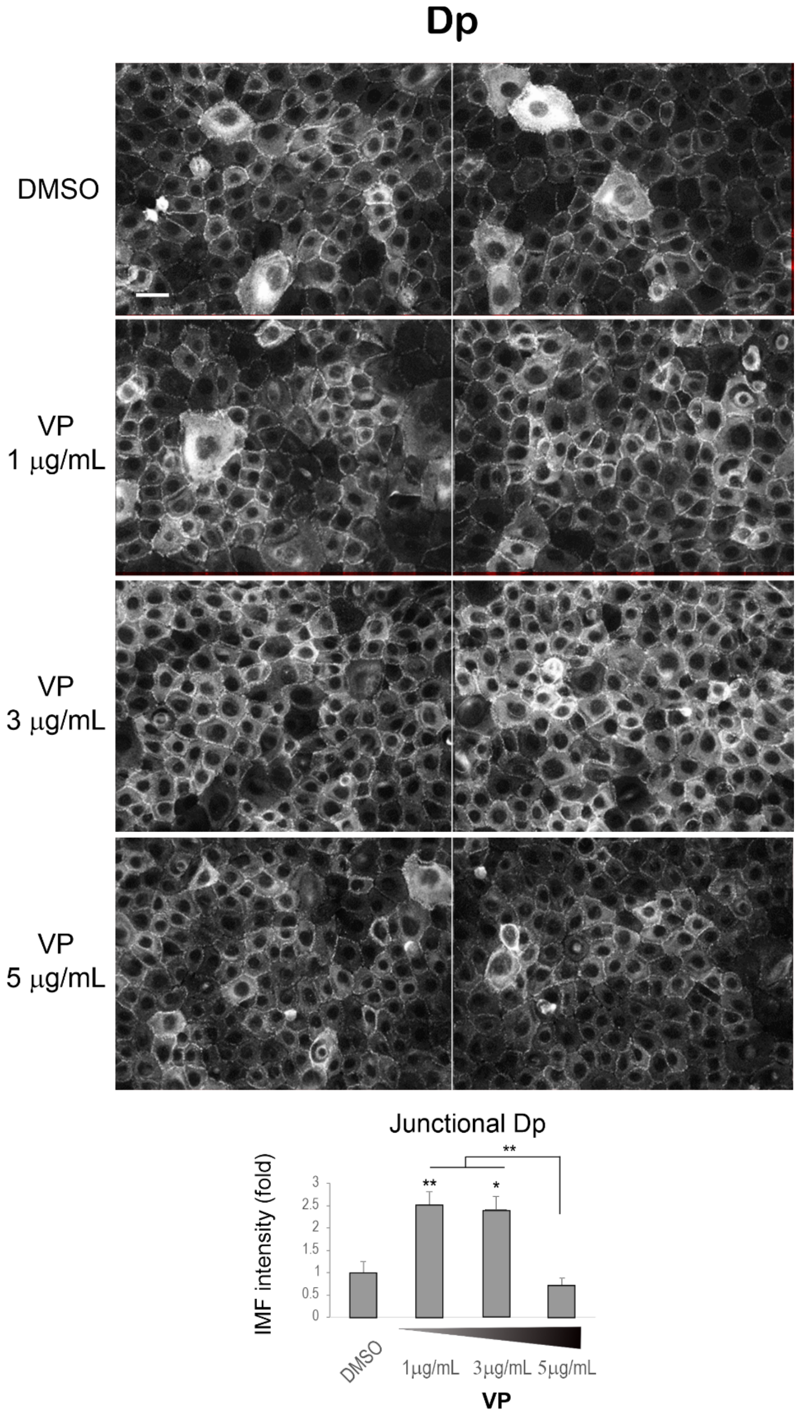


**Figure S4.** Immunofluorescence for Desmoplakin in N/TERT cells treated with VP. The epi-fluorescent microscopic images showed an elevated expression of Desmoplakin (Dp) in cells treated with VP at 1 and 3 μg/mL concentrations compared to cells treated with VP at 5 μg/mL or the DMSO vehicle control that showed no significant difference. Image quantitation was displayed underneath (n=5 fields/coverslips, Mean ± SEM, one-way ANOVA was used to determine the p values, *p<0.05, **p<0.01). Scale bar, 20 μm.
